# Discovery and characterization of SARS-CoV-2 reactive and neutralizing antibodies from humanized CAMouse^HG^ mice through rapid hybridoma screening and high-throughput single-cell V(D)J sequencing

**DOI:** 10.3389/fimmu.2022.992787

**Published:** 2022-09-23

**Authors:** Xi Yang, Hang Chi, Meng Wu, Zhenshan Wang, Qiaoli Lang, Qiuxue Han, Xinyue Wang, Xueqin Liu, Yuanguo Li, Xiwen Wang, Nan Huang, Jinhao Bi, Hao Liang, Yuwei Gao, Yongkun Zhao, Na Feng, Songtao Yang, Tiecheng Wang, Xianzhu Xia, Liangpeng Ge

**Affiliations:** ^1^ Institute of Bioengineering, ChongQing Academy of Animal Sciences, Chongqing, China; ^2^ Changchun Veterinary Research Institute, Chinese Academy of Agricultural Sciences, Changchun, China; ^3^ College of Veterinary Medicine, Jilin Agricultural University, Changchun, China; ^4^ Institute of Laboratory Animal Science, Chinese Academy of Medical Sciences (CAMS) and Comparative Medicine Center, Peking Union Medical College (PUMC), Beijing, China; ^5^ College of Wildlife and Protected Area, Northeast Forestry University, Harbin, China; ^6^ Food and Drug Inspection Laboratory, Administration for Drug and Instrument Supervision and Inspection, Beijing, China

**Keywords:** SARS-CoV-2, reactive and neutralizing antibodies, fully human antibody, antibody humanized mice, single-cell sequencing

## Abstract

The coronavirus disease 2019 pandemic has caused more than 532 million infections and 6.3 million deaths to date. The reactive and neutralizing fully human antibodies of severe acute respiratory syndrome coronavirus 2 (SARS-CoV-2) are effective detection tools and therapeutic measures. During SARS-CoV-2 infection, a large number of SARS-CoV-2 reactive and neutralizing antibodies will be produced. Most SARS-CoV-2 reactive and neutralizing fully human antibodies are isolated from human and frequently encoded by convergent heavy-chain variable genes. However, SARS-CoV-2 viruses can mutate rapidly during replication and the resistant variants of neutralizing antibodies easily survive and evade the immune response, especially in the face of such focused antibody responses in humans. Therefore, additional tools are needed to develop different kinds of fully human antibodies to compensate for current deficiency. In this study, we utilized antibody humanized CAMouse^HG^ mice to develop a rapid antibody discovery method and examine the antibody repertoire of SARS-CoV-2 RBD-reactive hybridoma cells derived from CAMouse^HG^ mice by using high-throughput single-cell V(D)J sequencing analysis. CAMouse^HG^ mice were immunized by 28-day rapid immunization method. After electrofusion and semi-solid medium screening on day 12 post-electrofusion, 171 hybridoma clones were generated based on the results of SARS-CoV-2 RBD binding activity assay. A rather obvious preferential usage of IGHV6-1 family was found in these hybridoma clones derived from CAMouse^HG^ mice, which was significantly different from the antibodies found in patients with COVID-19. After further virus neutralization screening and antibody competition assays, we generated a noncompeting two-antibody cocktail, which showed a potent prophylactic protective efficacy against SARS-CoV-2 in cynomolgus macaques. These results indicate that humanized CAMouse^HG^ mice not only provide a valuable platform to obtain fully human reactive and neutralizing antibodies but also have a different antibody repertoire from humans. Thus, humanized CAMouse^HG^ mice can be used as a good complementary tool in discovery of fully human therapeutic and diagnostic antibodies.

## Introduction

The coronavirus disease 2019 (COVID-19) pandemic has infected over 532 million people and led to loss of more than 6.3 million lives worldwide as of June 12, 2022 (https://www.who.int/).

Neutralizing antibodies are crucial components of the humoral immune system for preventing viral infections (1). These antibodies not only can block viral entry into host cells but also clear viral particles by using Fc-mediated effector functions (2). In addition to preventing infection of exposed individuals (3), treating COVID-19 and preventing progress to severe disease are potential benefits of passive immunization with neutralizing antibodies (4–6). Numerous efforts have been made to generate SARS-CoV-2 neutralizing antibodies. Several of these antibodies are emergency approved by the European Medicines Agency and Food and Drug Administration or undergoing phase III clinical trials (7).

Although antibody-mediated treatment has been successful, an increasing number of SARS-CoV-2 neutralizing antibodies have been reported to exhibit reduced effectiveness or loss of neutralizing activity to new strains of SARS-CoV-2 (8, 9). On the one hand, this is probably due to the fact that SARS-CoV-2 neutralizing antibodies isolated from human are frequently encoded by convergent heavy-chain variable genes that share similar binding modes and footprints (10, 11). As a result of limited diversity, the focused antibody responses in human could result in antibody escape mutations. On the other hand, almost all SARS-CoV-2 neutralizing antibodies are isolated from patients with COVID-19. During SARS-CoV-2 infection, a large number of SARS-CoV-2 reactive and neutralizing antibodies will be produced (12, 13). Meanwhile, SARS-CoV-2 viruses can mutate rapidly during replication and the resistant variants of neutralizing antibodies easily survive and evade the immune response. Therefore, these new challenges and questions require additional tools to develop different kinds of fully human antibodies to compensate for current deficiency.

CAMouse^HG^ is a novel immunoglobulin (IG) humanized mouse model (http://cn.cnmab.com). To solve the problem of human immunoglobulin genes’ incompatibility during mouse B-cell development, a nucleic acid molecule comprising thirty-two human IGH, 22 human IGK and 16 IGL genes (14) were inserted in the random region of the CAMouse^HG^ genome by using an efficient method of cell fusion (15) while endogenous mouse IGH and IGK genes, except for mouse IgM and its control elements, were inactivated (16). CAMouse^HG^ mice express human-mouse chimeric IgM (human V region with mouse C regions) and fully human IgG. We hypothesized that CAMouse^HG^ mice could have a different antibody repertoire from humans.

High-throughput single-cell V(D)J sequencing technology is a powerful tool used to investigate large-scale antibody gene expression profiles at single-cell resolution (17, 18). Diverse antibodies are capable of recognizing and fighting multiple pathogen invasions to provide long-term protection. The present study aimed to characterize the SARS-CoV-2 reactive and neutralizing antibody repertoire of humanized CAMouse^HG^ mice. By using the SARS-CoV-2 RBD protein as an immunogen, we developed a protocol for rapid identification of fully human SARS-CoV-2 antibodies through hybridoma screening. The antibody repertoire was revealed with high-throughput single-cell V(D)J sequencing approach. First, five CAMouse^HG^ mice were immunized in 1 week interval with the RBD protein by 28-day rapid immunization method. After serum antibody titer analysis using ELISA, the spleen cells of 2 CAMouse^HG^ mice with high antibody titer were isolated and fused with myeloma cells to obtain hybridoma cells. In this study, 1920 monoclonal hybridoma cell lines were generated with further semi-solid medium screening on day 12 post-electrofusion. Then, we generated 171 hybridoma clones after SARS-CoV-2 RBD binding activity assay. Except for one clone lost during culture, 170 RBD-reactive hybridoma cell clones were mixed in equal proportions and used for single-cell V(D)J sequencing. The majority of cells were IgG1-secreting hybridomas (66.98%) and IgG3-secreting hybridomas (4.51%), and only 28.51% were IgM-secreting hybridomas. The predominant lineage of antibodies derived from CAMouse^HG^ mice utilized IGHV6-1 paired with IGKV3-20, IGKV1-5, and IGKV3-15, which significant differed from antibodies in patients with COVID-19. Among 171 hybridoma clones, 53 blocked the interaction of RBD with ACE2 receptors. The top 20 clones with the highest RBD-ACE2 blocking activity were evaluated for live virus neutralization assay. Finally, eight hybridoma clones capable of neutralizing the live virus were obtained. After 5’ RACE (5’ rapid amplification of cDNA ends) sequencing and recombinant expression, five recombinant neutralizing antibodies were obtained. Two antibodies (11-2G and 18-4A) were selected as a two-antibody cocktail based on antibody competition assay data. The 11-2G and 18-4A showed different binding modes towards SARS-CoV-2 spike protein in protein–protein docking analysis, and efficiently bind to SAR-CoV-2 RBD with affinity of 0.451 nM and 0.234 nM, respectively. The 11-2G/18-4A cocktail showed potent prophylactic efficacy in SARS-CoV-2-infected cynomolgus macaques.

Our results indicate that humanized CAMouse^HG^ mice not only provide a valuable platform to obtain fully human reactive and neutralizing antibodies but also have a different antibody repertoire from humans. Hence, these mice can be used as a good complementary tool in the discovery of fully human therapeutic and diagnostic antibodies.

## Materials and methods

### Ethics and biosafety statement

All experiments involving cynomolgus macaques were approved by the Institutional Animal Care and Use Committee of Laboratory Animal Centre (Assurance Number: IACUC-DWZX-2020-030). All tests for the infectious SARS-CoV-2 virus were performed in the Biosafety Level 3 (BSL-3) Laboratory.

### Cell lines, virus and animals

Vero E6 and HEK293T-hACE2 cells were grown in DMEM (Gibco, San Diego, CA, USA) supplemented with 10% fetal bovine serum (FBS) and 1% penicillin–streptomycin (Gibco, San Diego, CA, USA). The wildtype SARS-CoV-2 strain (BetaCoV/Beijing/IME-BJ01/2020, GWHACAX01000000) was isolated from patients with COVID-19 in China. The authentic SARS-CoV-2 was passaged in Vero E6 cells and titrated in the same cell line. Cynomolgus macaques with similar age and body weight (6.5–7.9 kg) were selected.

### Generation of mAbs from CAMouse^HG^ mice

The animal studies were approved by the Institutional Animal Care and Use Committee at Chongqing Academy of Animal Sciences. CAMouse^HG^ mice were subjected to multiple subcutaneous points primed with the SARS-CoV-2 RBD protein (Sino Biological, Beijing, China) (100 μg/mouse) in complete Freund’ adjuvant (Sigma-Aldrich) plus 25 μg of CpG (Sangon Biotech, China) and 1% (v/v) Alhydrogel (vac-alu-50, *In vivo*gen). The mice were then successively boosted with the SARS-CoV-2 RBD protein in incomplete Freund’s adjuvant (Sigma-Aldrich) plus CpG and Alhydrogel at 1 week interval. Four days after the last injection, spleen cells were isolated and mixed with SP2/0 cells at a ratio of 1:5 to obtain hybridoma cells by electrofusion. Electrofusion was performed using BTX ECM2001 (BTX, San Diego, CA) by application a single 40-μs pulses at 900 V. The fused cells were added into ClonaCell™-HY Medium D (STEMCELL Technologies) to a final concentration of 0.9 × 10^7^ cells/mL. and cultured at 37°C in a 5% CO2 incubator. After 8 days of culture, cell clones were picked and seeded into a 96-well plate. Cell supernatant was collected after 3-4 days culture for the further analysis.

### ELISA analysis of antibody binding to SARS-CoV-2 RBD

ELISA plates (Hyclone, Shanghai, China) were coated with SARS-CoV-2 RBD-mFc (SinoBiological, Beijing, China) at 4°C overnight. After washing with phosphate buffer saline containing 0.05% Tween-20 (PBST), the plates were blocked with 1% BSA (Sigma-Aldrich, shanghai, China) in PBST at room temperature for 1 h followed by three washing steps. Serially diluted purified antibodies, mouse serum samples, or hybridoma culture supernatants were allowed to bind to the plates at 37°C for 2 h. Antibody binding responses were detected using Mouse Anti-Human IgG Fab-HRP (GenScript; diluted 1: 5000 in 1% PBST) at 37°C for 2 h, followed by three washing steps. The plates were developed with 3,3’,5,5’-tetramethylbenzidine (TMB) (Solarbio, Beijing, China) as a substrate. The reaction was stopped with 2.5 M H_2_SO_4_, and the absorbance of each well was monitored at 450 nm.

### Single-cell antibody repertoire library preparation

Equal amounts of ELISA-positive hybridoma cell clones bound to SARS-COV-2 RBD were encapsulated with DNA-barcoded gel beads by using a 10× chromium controller. Cells were counted using the Countstar Automated Cell Counter and normalized to 1 × 10^4^ cells in reverse transcription (RT) mix. Target enrichment RT and library preparation were done according to the manufacturer’s instructions using the following kits: Chromium Single Cell 5’ Library & Gel Bead Kit (PN-1000014), Chromium Single Cell V(D)J Enrichment Kit, Human B Cell (PN-1000016), Chromium Single Cell A Chip Kit (PN-1000009) and Chromium i7 Multiplex Kit (PN-120262). Single-cell antibody repertoire libraries were sequenced on an Illumina NovaSeq 6000 system (Illumina RTA Version: V3.4.4). Sequencing revealed that 4287 cells were recovered. The processing of raw data resulted in 88,514 reads per hybridoma cell. The reads were then filtered, processed and assembled into contigs for final analysis. The 10× Genomics CellRanger pipeline (version 3.0.1) was used for sequencing data processing. Refdata-cellranger-vdj-GRCh38-alts-ensembl-3.1.0 were used as reference.

### ACE2 blocking assay

SARS-CoV-2 RBD-mFc (SinoBiological, Beijing, China) at 2 µg/mL was loaded on ELISA plates (Hyclone, Shanghai, China) at 4°C overnight. The plates were washed three times and then blocked with 1% BSA in PBST at RT for 1 h. After washing with PBST, 25 μL of serially diluted purified antibodies, mouse serum samples or hybridoma culture supernatants were mixed with 25 μL of the human ACE2-his protein (1 µg/mL) (Novoprotein, Shanghai, China). The samples were then added with 50 μL of the mixtures and incubated at 37°C for 2 h. After washing, ACE2 binding responses were detected using Anti-6×His tag-HRP (Abcam; diluted 1: 5000 in 1% PBST) at 37°C for 2 h. The plates were washed three times with PBST and then developed with 3,3’,5,5’-tetramethylbenzidine (TMB) (Solarbio, Beijing, China) as a substrate. The reaction was stopped with 2.5 M H_2_SO_4_, and the absorbance of each well was monitored at 450 nm. IC50 values were calculated by three-parameter fitting using GraphPad Prism 6 software.

### Production of recombinant human mAbs

DNA fragments encoding the antibody variable regions of hybridoma cells were cloned and sequenced with 5 ‘RACE method by a commercial sequencing service (Genewiz, Suzhou, China). Recombinant human IgG1 mAbs were synthesized using DNA fragments encoding heavy-chain and light-chain genes and their signal sequence by Genewiz, Inc. (Suzhou, China) and cloned into modified pcDNA3.4 vectors that contain human constant regions of IgG1 or light chains. HEK293F cells were transiently cotransfected with pairs of the heavy-chain and light-chain expression vectors by using EZ cell transfection reagent (Life-iLab, Shanghai, China) according to the instructions’ manual. After 7 days of culture, the cell supernatants were harvested and purified using MabSelect SuRe™ LX (GE Healthcare, Sweden).

### Antibody binding competition assay

Antibody binding competition assay was performed using Bio-Layer Interferometry (BLI) on Octet QK instrument (ForteBio). The first mAb (30 μg/mL) was immobilized onto the anti-human IgG Fc capture (AHC) (Fortebio, China). After washing with PBS, the biosensor tips were immersed into a well containing the wildtype SARS-CoV-2 RBD Protein (SinoBiological, Beijing, China) at a concentration of 51.5 μg/mL and loaded into a well containing the secondary mAb at a concentration of 30 μg/mL.

### Epitope distribution analysis

The 3D structure of the fragment antigen-binding (Fab) regions of antibodies were obtained by homology modelling using the MODELLER program package (19). The structure of the SARS-CoV-2 spike protein (PDB ID:7PTH) was retrieved from Protein Data Bank (PDB). Protein–protein docking was performed with ZDOCK-server to obtain antibody–antigen complexes (20), generating 2000 docking solutions. Among them, the best 7 were selected based on docking scores. PyMol software was used to visualize the results.

### Surface plasmon resonance (SPR) analysis

SPR measurements were done by a commercial sequencing service company (YanGene, Wuhan, China) on a Biacore T200. Briefly, mAbs were captured on the chip and gradient concentrations of the SARS-CoV-2 RBD protein were then passed over the chip surface. Affinity was calculated using Biacore T200 evaluation software.

### Pseudovirus-based neutralization assay

HIV-based SARS-CoV-2 pseudoviruses were generated by co-transfection of HEK293T cells with the HIV backbone pNL4-3.Luc.RE and the recombinant plasmid pcDNA3.1-SΔ19 encoding the spike protein of the wildtype SARS-CoV-2 strain (Wuhan-Hu-1, GenBank: MN908947.3) with D614G mutation where the last 19 amino acids of the cytoplasmic tail were excluded. In brief, 3-fold serially diluted antibodies were incubated with SARS-CoV-2 pseudovirus at 37°C for 1 h. Approximately 2 × 10^4^ HEK293T-hACE2 cells were added into the mixture. After incubation at 37°C for 48 h, the luciferase activity of cell lysis was measured using a One-Lumi Firefly Luciferase Assay Kit (Beyotime, Shanghai, China). Inhibition percentage was calculated as the relative luminescent units (RLUs) of sample wells compared with viral control wells. Half maximal inhibitory concentration (IC_50_) values were analyzed using GraphPad Prism 8.0 software (GraphPad Software Inc., USA).

### Authentic SARS-CoV-2 neutralization assay

Authentic SARS-CoV-2 neutralization assay was performed based on cytopathic effect (CPE) assay in the approved Biosafety Level 3 facility. The cultural supernatant of hybridoma cells or 3-fold serially diluted antibodies in 96-well plates were incubated with equal volume of 100 TCID_50_ of authentic wildtype SARS-CoV-2 (BetaCoV/Beijing/IME-BJ01/2020) at 37°C for 1 h. The mixture was added with 1 × 10^4^ Vero E6 cells and incubated at 37°C for 3 days. Cells infected with 100 TCID_50_ of authentic SARS-CoV-2 and without the virus were applied as infected and uninfected control, respectively. Virus back-titration was performed simultaneously. The cytopathic effect (CPE) of each well was observed daily and recorded on day 3 post infection. For the hybridoma cells supernatant, the wells without CPE were determined as neutralization. For the antibodies, the 50% maximal inhibitory concentration (IC_50_) values were calculated by Reed–Muench method.

### Antibody treatment and SARS-CoV-2 challenge of cynomolgus macaques

Cynomolgus macaques were randomly divided into two groups for evaluation of the prophylactic efficacy of the 11-2G/18-4A cocktail. Three cynomolgus macaques were intravenously (i.v.) injected with 50 mg/kg antibody cocktail (composed of 18-4A and 11-2G, 1:1 ratio), while the two other cynomolgus macaques received 50 mg/kg IgG1 isotype control one day before SARS-CoV-2 infection. All cynomolgus macaques were intranasal (i.n.) challenged with 1.0 × 10^5.7^ TCID_50_ of wildtype SARS-CoV-2 (BetaCoV/Beijing/IME-BJ01/2020). Nasal, throat and anal swabs were collected at 2-, 4- and 6-days post-infection. At 7 days post-infection, cynomolgus macaques were euthanized. The lungs (both left lobe and right lobe) and tracheas from cynomolgus macaques of each group were obtained. A portion of the above tissues was homogenized for the detection of SARS-CoV-2 genomic RNA and subgenomic RNA through Real-Time Quantitative Reverse Transcription PCR (qRT-PCR) assay as previously described (21). A portion of the lungs were fixed in 4% formaldehyde and embedded with paraffin for histopathology evaluation by hematoxylin and eosin (H&E) assay.

### Statistical analysis

All statistical analyses were performed using GraphPad Prism version 6. Statistical significance was defined as *p < 0.05, **p < 0.01, ***p < 0.001, ****p < 0.0001.

## Results

### Rapid isolation of anti-SARS-CoV-2 RBD antibodies from CAMouse^HG^

**Figure 1:**
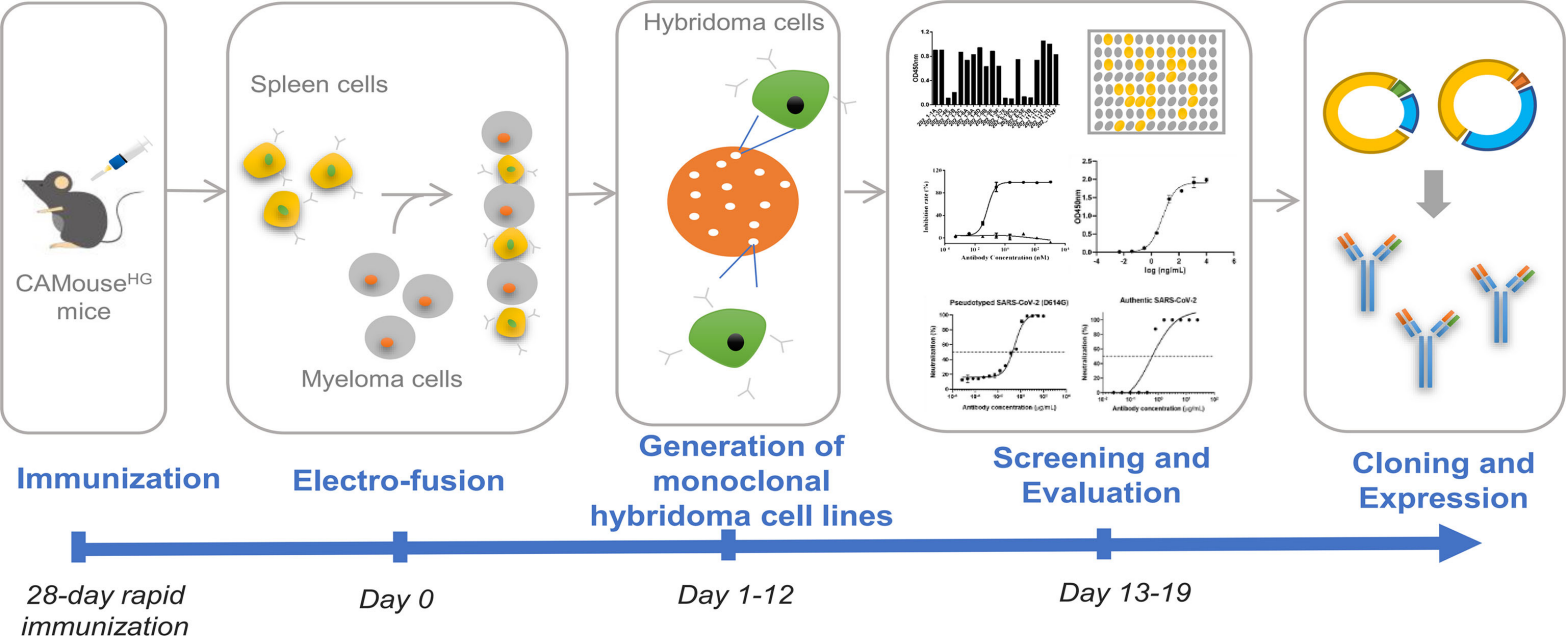
Integrated workflow for rapid isolation of anti-SARS-CoV-2 RBD antibodies from CAMouse^HG^ mice.

We utilized CAMouse^HG^ mice, which encode the human immunoglobulin repertoire, to identify fully human monoclonal antibodies (mAbs) targeting the RBD protein of SARS-CoV-2. As shown in (**Figure 1**), five CAMouse^HG^ mice were immunized in 1 week interval with SARS-CoV-2 RBD to develop a 28-day rapid immunization scheme. After the serum of mice was collected for ELISA titer detection, the spleen cells of two CAMouse^HG^ mice with high antibody titer were isolated and fused with myeloma cells to obtain hybridoma cells by electrofusion. The day of hybridoma electrofusion was marked as Day 0. The fused cells were screened and cultured by semi-solid medium to generate monoclonal hybridoma clones (Days 1–12). In this study, 1920 hybridoma monoclonal clones were selected for further screening and evaluation (Days 13–19). The supernatant was collected for ELISA analysis of antibody binding to SARS-CoV-2 RBD. The results showed that 171 hybridoma clones strongly bound to SARS-CoV-2 RBD (**Supplementary Table 1**). Fifty-three of them blocked the interaction of RBD with ACE2 receptors (**Supplementary Table 2**). The top 20 clones with the highest RBD–ACE2 blocking activity were evaluated for live virus neutralization assay. Finally, eight hybridoma clones capable of neutralizing the live virus were obtained. After 5’ RACE sequencing of antibody variable regions, six kinds of mAbs were obtained. The heavy-chain and light-chain genes of them were cloned into a human IgG1 isotype backbone to express recombinant antibodies in HEK 293F cells. Finally, five recombinant antibodies showed inhibitory activity to SARS-CoV-2 virus and named as 4-1B, 4-2D, 11-2G, 15-11F and 18-4A.

### Single-cell antibody repertoire sequencing and analysis of RBD-reactive hybridoma cells from CAMouse^HG^ mice

**Figure 2:**
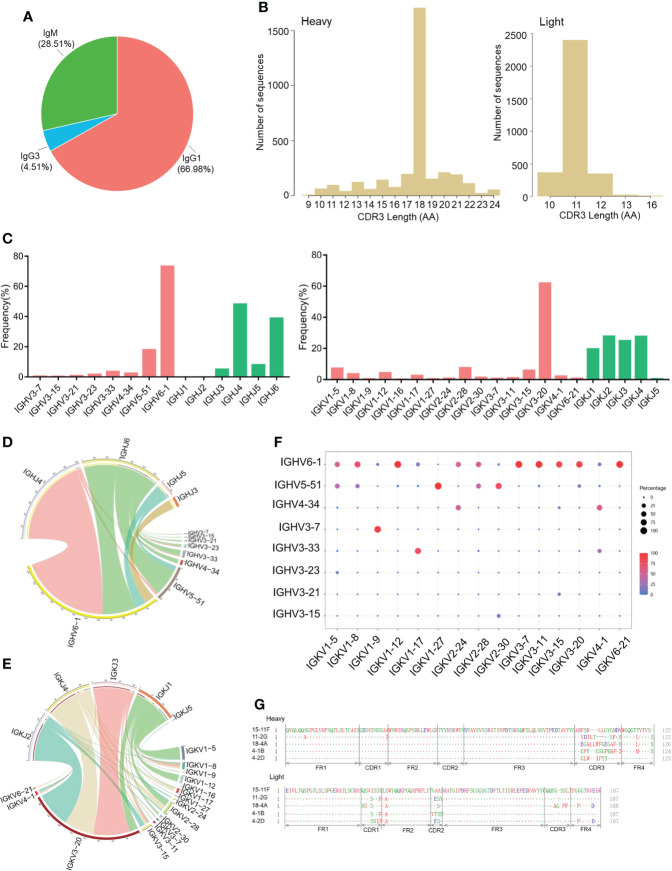
Antibody repertoire features of RBD-reactive hybridoma cells derived from CAMouse^HG^ mice. **(A)** Isotype usage in single-cell repertoire sequencing data of hybridoma clones specifically recognized SARS-COV-2 RBD. **(B)** CDR3 AA lengths of heavy and light chains. **(C)** Human V and J genes of heavy- and light-chain utilization. Circos plots comparing VJ gene association of heavy **(D)** and light chains **(E)**. VJ combinations are linked based on their relative frequency. **(F)** Paired antibody repertoire. Circle size and color correspond to the number of heavy and light chains present. **(G)** Amino acid sequence alignment of the five SARS-COV-2 neutralizing mAbs isolated from CAMouse^HG^ mice.

To investigate the antibody repertoire of SARS-CoV-2 RBD-reactive hybridoma cells, we screened 170 hybridoma cell clones based on the binding activity of the cell supernatant to the SARS-CoV-2 RBD protein. The clones were then used for single-cell sequencing analysis (1 out of 171 RBD-reactive hybridoma cell clones was lost during culture). A single-cell library was prepared using the Chromium Single Cell 5’ Library & Gel Bead Kit and Chromium Single Cell V(D)J Enrichment Kit. The results of isotype usage analysis showed that the majority of cells were IgG1-secreting hybridomas (66.98%) and IgG3-secreting hybridomas (4.51%), and only 28.51% were IgM-secreting hybridomas (**Figure 2A**). CDR3 AA lengths of heavy- and light-chain variable regions were diverse (**Figure 2B**). In the heavy-chain genes of RBD-reactive hybridoma cells derived from CAMouse^HG^ mice, the highest frequency of use was IGHV6-1 (73.2%), followed by IGHV5-51 (17.9%) (**Figure 2C**). This finding is totally different from the antibodies isolated from patients with COVID-19, which showed strong enrichment in the usage of the IGHV3 family (22–24). In addition, IGHJ6 and IGHJ4 were used most frequently in CAMouse^HG^ mice. IGKV3-20 had the highest frequency of use, and IGKJ1, IGKJ2, IGKJ3 and IGKJ4 showed a high frequency of use (**Figure 2C**). Similarly, a significant overexpression of IGKV3-20 and IGHJ6 was found in antibodies isolated from patients with COVID-19 (25).

We then analyzed the pairing ratio of IGHV with IGHJ or IGKV in the RBD-reactive hybridoma cells of CAMouse^HG^ mice. As shown in (**Figure 2D**), IGHJ6 paired with a wide range of IGHV but rarely paired with IGHV6-1, which was mainly paired with IGHJ4. The Circos plots of the V-J segments showed that the most frequently used light-chain V gene IGKV3-20 widely paired with IGKJ1-4 (**Figure 2E**). IGHV6-1 and IGHV5-51 were shown to pair with a wide range of IGKV in RBD-reactive hybridoma cells derived from CAMouse^HG^ mice, while other IGHVs such as IGHV3-7, were found only in combination with a small number of IGKV (**Figure 2F**).

We also characterized five SARS-COV-2 neutralizing mAbs derived from CAMouse^HG^ mice. The sequence diversity of neutralizing mAbs is presented in (**Figure 2G**). Based on the analysis of antibody sequences, these neutralizing mAbs revealed a significant trend in antibody repertories. Heavy and light chains were derived from the same IGHV6-1 and IGKV3-20 germline gene. These mAbs possessed low mutation rates in heavy- and light-chain variable regions, which were 0–0.3% and 1.4–1.7%, respectively (**Table 1**).

**Table 1:** Characterization of SARS-COV-2 neutralizing mAbs isolated from CAMouse^HG^ mice.

mAbs	Heavy chain				Light chain		
	V gene	D gene	J gene	mutation rate (%)	V gene	J gene	mutation rate (%)
**4-1B**	IGHV6-1	IGHD3-10	IGHJ3	0.3	IGKV3-20	IGKJ4	1.4
**4-2D**	IGHV6-1	IGHD4/OR15-4a	IGHJ6	0	IGKV3-20	IGKJ3	1.4
**11-2G**	IGHV6-1	IGHD3-9	IGHJ4	1.3	IGKV3-20	IGKJ4	1.4
**15-11F**	IGHV6-1	IGHD3-16	IGHJ6	1.3	IGKV3-20	IGKJ4	1.7
**18-4A**	IGHV6-1	IGHD3-10	IGHJ4	0	IGKV3-20	IGKJ3	1.7

Overall, some similarities existed between CAMouse^HG^ mice and human, but the differences were more important. Our data suggest that humanized CAMouse^HG^ mice have a different antibody repertoire from humans and can be used as a good complementary tool in the discovery of fully human therapeutic and diagnostic antibodies.

### Selection of a noncompeting two-antibody cocktail

The combined use of two or more antiviral antibodies targeting different epitopes can reduce therapeutic efficacy and drug resistance risks. Therefore, we aimed to identify a noncompeting mAbs pair.

**Figure 3:**
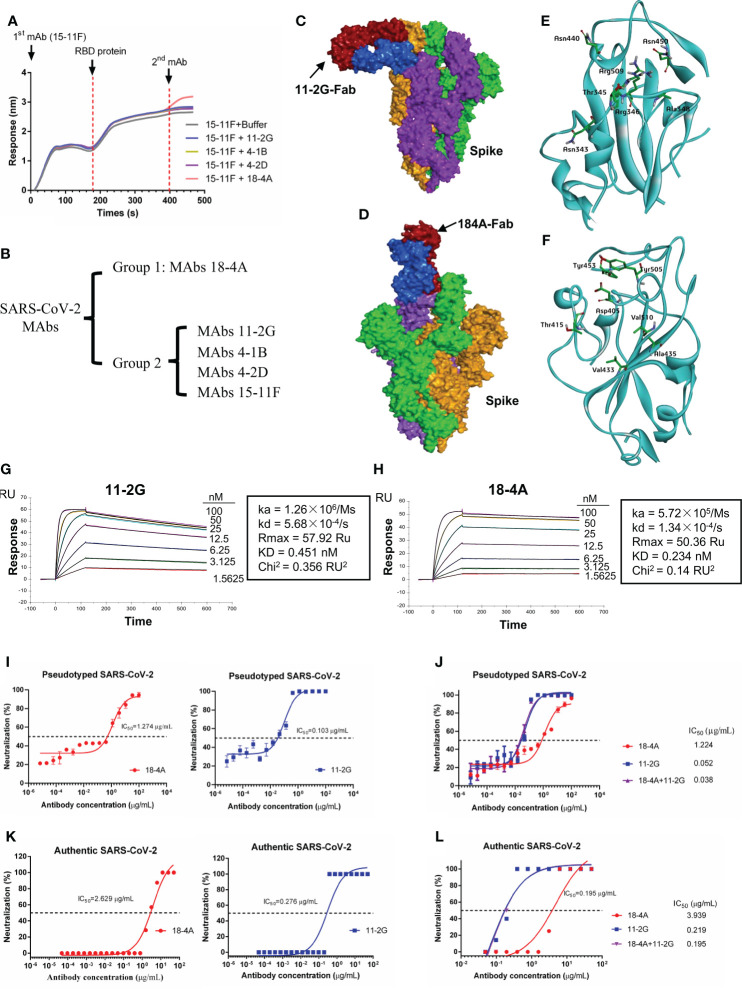
Characterization of mAbs against SAR-CoV-2 RBD. **(A)** Antibody binding competition assay. Immobilized first mAb (30 μg/mL) was mixed with SAR-CoV-2 RBD (51.5 μg/mL), and secondary mAb (30 μg/mL) was used to compete for binding. **(B)** Grouping of mAbs. Group 1, MAb 18-4A; group 2, the other mAbs. Structural analyses of SARS-CoV-2 spike and Fab complex. **(C)** Structure of SARS-CoV-2 spike (PDB code: 7TPH) in complex with 11-2G-Fab. **(D)** Structure of SARS-CoV-2 spike (PDB code: 7TPH) in complex with 18-4A-Fab. The different subunits of the spike protein are colored green, orange, purple; two Fab domains of antibodies are colored blue and red, respectively. Binding details between and SARS-CoV-2 spike and 11-2G-Fab **(E)** or 18-4A-Fab **(F)** are presented with amino acids from SARS-CoV-2 spike, which forms hydrophobic or hydrogen bond interactions with amino acids from 11-2G-Fab. **(G, H)** Analysis of affinity of 11-2G and 18-4A for the SARS-CoV RBD protein. **(I)** Neutralizing activity of 11-2G and 18-4A against SARS-CoV-2 pseudoviruses. **(J)** Neutralizing activity of 11-2G/18-4A cocktail (1:1) against SARS-CoV-2 pseudoviruses. **(K)** Neutralizing activity of 11-2G and 18-4A against authentic SARS-CoV-2. **(L)** Neutralizing activity of 11-2G/18-4A cocktail (1:1) against authentic SARS-CoV-2.

Antibody competition assays were performed to investigate the epitopes of mAbs. The results showed that 18-4A produced the highest additional signal, suggesting that 18-4A can recognize an epitope distinct from the binding site of the first immobilized antibody (15-11F). By contrast, incubation with 11-2G, 4-1B or 4-2D produced only a slight increase in signals, indicating that these mAbs can recognize similar epitopes on RBD with 15-11F (**Figure 3A**). Based on the above antibody competition assay data, these antibodies were divided into two antibody groups (**Figure 3B**). We selected 11-2G from group1 and 18-4A from group 2 for pairing.

To gain a more comprehensive understanding of how 11-2G and 18-4A bind to the spike protein of SARS-CoV-2, we analyzed the structure of SARS-CoV-2 spike in complex with the fragment antigen-binding (Fab) regions of 11-2G or 18-4A by protein–protein docking. The structure of Fab bound to the SARS-CoV-2 spike is shown in (**Figures 3C, D**). The RBD residues including Arg343, Arg345, Arg346, Arg 348, Asn440, Asn450 and Arg509 formed hydrophobic or hydrogen bond interactions with amino acids with 11-2G (**Figure 3E**). By contrast, Asp405, Thr415, Val433, Ala435, Tyr453, Tyr505 and Val510 are targeted *via* hydrophobic or hydrogen bond with 18-4A (**Figure 3F**). For 18-4A, the binding site mainly focused on 405-510 of RBD, which widely overlap the binding site of ACE2 (26). For 11-2G, the binding site comprises predominantly 2 segments: 343-348 and 440-509 of the RBD, which largely outside of the ACE2 sits. These structural data for the two mAbs, which were selected from CAMouse^HG^ mice, indicate the different modes of recognition for antibodies 11-2G and 18-4A.

To characterize the activity of 11-2G and 18-4A, we performed binding assays measuring the ability of the purified antibody samples to bind to the RBD of SARS-CoV-2. Analysis of the data obtained from SPR demonstrated that 11-2G and 18-4A could efficiently bind to SAR-CoV-2 RBD with affinity of 0.451 nM (**Figure 3G**) and 0.234 nM (**Figure 3H**), respectively. The recombinant antibody 18-4A and 11-2G demonstrated neutralizing activity against pseudotyped (**Figure 3I**) and authentic SARS-CoV-2 (**Figure 3K**). Of note, the 11-2G/18-4A cocktail displayed higher neutralization potencies against SARS-CoV-2 pseudovirus than that of parental mAbs (**Figure 3J**). Similar neutralization trends were shown in the authentic SARS-CoV-2 infection system (**Figure 3L**). The 11-2G/18-4A cocktail neutralized the pseudotyped and authentic SARS-CoV-2 with half maximal inhibitory concentration (IC50) values of 0.038 and 0.195 μg/mL (0.25 and 1.3 nM), respectively. To detect whether the antibodies recognized epitopes on the natural protein, 293T cells were transiently transfected with plasmids to express SARS-CoV-2 Spike on the surface. Flow cytometry was performed using the recombinant antibodies 11-2G (4 μg/mL) or 18-4A (4 μg/mL) as primary antibodies, and Goat anti-Human IgG (H+L) Cross-Adsorbed Secondary Antibody, Alexa Fluor™ 633 (invitrogen, 1:500) as detected antibody to stain cells. Both recombinant antibodies 11-2G and 18-4A were able to recognize epitopes on the natural spike protein (**Supplementary Figure S1**).

### 11-2G/18-4A cocktail showed potent prophylactic efficacy in SARS-CoV-2-infected cynomolgus macaques

**Figure 4:**
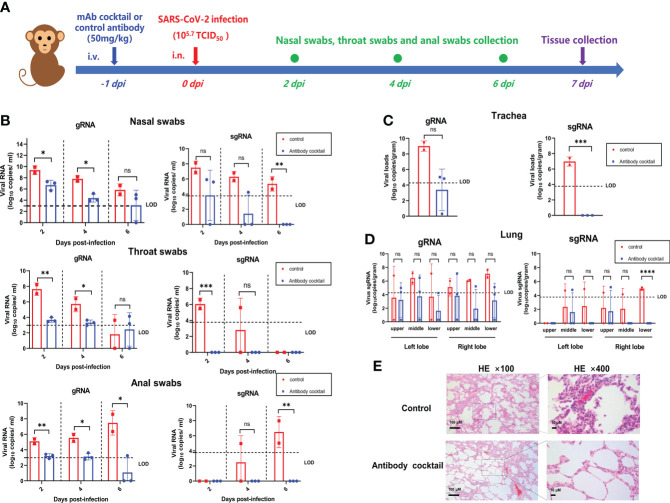
The 11-2G/18-4A cocktail showed potent prophylactic efficacy in SARS-CoV-2-infected cynomolgus macaques. **(A)** Schematic diagram of antibody treatment and SARS-CoV-2 challenge in cynomolgus macaques. **(B)** Nasal, throat and anal swabs were collected at 2, 4, 6 days post-infection. **(C)** Trachea and **(D)** Lungs (both left lobe and right lobe) were obtained at 7 days post-infection. Viral load was assessed by detection of SARS-CoV-2 genomic RNA and subgenomic RNA using qRT-PCR. Data are shown as mean ± SD. An unpaired Student’s t-test (two-tailed) was used for statistical analysis, and the relevant p-values are indicated (not indicated in graph; *p < 0.05, **p < 0.01, ***p < 0.001, ****p < 0.0001). **(E)** H&E staining of lung tissues. ns, not significant.

To evaluate the *in vivo* efficacy of the 11-2G/18-4A cocktail, we performed prophylactic experiments in an infection model of cynomolgus macaques (**Figure 4A**). After intranasal challenge with SARS-CoV-2, the levels of viral genomic RNA (gRNA) and subgenomic RNA (sgRNA) in swabs from the antibody-cocktail treated animals were lower or undetectable than the control antibody-treated animals at day 2, 4 and 6 post infection. Of note, sgRNA in throat swabs and anal swabs from the antibody cocktail-treated animals was undetectable from 1 dpi to 6 dpi, and no viral sgRNA were detected in the nasal, throat as well as anal swabs on day 6 post infection, indicating the absence of infectious viruses. This result demonstrated the antibody cocktail treatment successfully inhibited SARS-CoV-2 secretion in upper respiratory tract as well as fecal viral shedding (**Figure 4B**). On day 7 post infection, the antibody cocktail-treated animals displayed reduced viral gRNA in trachea and lung lobes compared with that in the control group. More importantly, no infectious viruses were detectable from cocktail-treated animals while high levels of viral sgRNA were detected from the control group, indicating that the 11-2G/18-4A cocktail was highly effective in eliminating viral replication in the upper and lower respiratory tracts of SARS-CoV-2-infected cynomolgus macaques (**Figures 4C, D**). Further lung histopathology evaluation showed that control animals developed moderate-to-severe pneumonia characterized by obvious diffuse alveolar damage, vanishment of pulmonary alveolar, congestion and interstitial lymphocytic inflammatory infiltrate. By contrast, the 11-2G/18-4A cocktail treatment largely alleviated lung damage, leading to significantly reduced pathological condition than that of the infection group (**Figure 4E**). Altogether, the 11-2G/18-4A cocktail showed a potent prophylactic protective efficacy against SARS-CoV-2 in cynomolgus macaques.

## Discussion

Neutralizing antibodies are crucial components of the humoral immune system for preventing viral infections. With the continuing emergence of resistant variants, additional traditional antibody–drug development methodologies that isolated antibodies from human, we need more tools to develop different kinds of fully human antibodies. In this study, we utilized humanized CAMouse^HG^ mice to develop a rapid antibody discovery method, analyzed the antibody repertoire of hybridoma cells of immunized CAMouse^HG^ mice and demonstrated that CAMouse^HG^ mice can be used as a good complementary tool in the discovery of fully human therapeutic and diagnostic antibodies.

CAMouse^HG^ mice are humanized transgenic mice with 70 kinds of human IgG variable and constant regions as well as inactivated mouse IgG heavy and light chains. Compared with antibodies isolated from patients with COVD-19, humanized CAMouse^HG^ mice were immunized repeatedly with immunogens combined with adjuvants; as such, researchers will no longer need to contact patients. Here, we elucidated the antibody response of hybridoma cells derived from CAMouse^HG^ mice by using SARS-CoV-2 reactive and neutralizing antibody screening combined with single-cell antibody repertoire sequencing. Similar to wild-type mice, CAMouse^HG^ mice can produce a high amount of IgG antibodies at 28 days after immunization. As shown in (**Figure 2**), after 28 days of rapid immunization with the SARS-CoV-2 RBD protein, 71.4% of SARS-CoV-2 reactivity antibodies were IgG type.

Single-cell sequencing was also used to discover SARS-CoV-2 antibodies from patients with COVID-19 (27–29). We therefore compared the germline profiles from CAMouse^HG^ single-cell repertoire data to human single-cell repertoire data. In COVID-19 infected persons, IGHV3-30, IGHV3-30-3, IGHV3-33 and IGHV5-51 were used most frequently (27, 30, 31). The usage of IGKV and IGHJ genes was observed very similar trends between human and CAMouse^HG^ mice. IGKV3-20 and IGHJ6 were highly expressed in both of them. However, different trends in IGHV gene usage were found between them. A rather obvious preferential usage of IGHV6-1 family was detected in CAMouse^HG^ mice (**Figure 2**).

However, there are also some limitations in this study. The antibody repertoire of hybridoma cells derived from unimmunized mice was not detected in this study. As a result, it is not clear whether this preferential usage of IGHV6-1 family in SARS-CoV-2 reactive and neutralizing antibodies from humanized CAMouse^HG^ mice is due to immunogen or the baseline antibody repertoire of CAMouse^HG^ mice. But in previous study, CAMouse^HG^ mice were immunized with peptide antigen of GPC3 (glypican-3). Five fully human anti-GPC3 antibodies generated in this study were originated from IGHV3-21 or IGHV3-7 (32). It suggests that the preferential usage of IGHV6-1 family in our study is likely due to immunogen specificity. We also tested the capacity of the antibody cocktail to neutralize current variants present in the population. SARS-CoV-2 Omicron variants has become the current predominant strain. As same as many SARS-CoV-2 neutralizing antibodies, including some clinically approved antibodies, our current selected antibodies have shown to exhibit strongly reduced neutralizing activity against Omicron variant pseudoviruses. The reasons for this are multiple. On the one hand, the epitope region recognized by antibody usually contains few amino acid residues. As such rapid viral mutation of SARS-CoV-2, it is also easy to cause neutralizing antibodies resistance regardless of the sources of antibodies. More important, in this study, we immunized CAMouse^HG^ mice with RBD protein of wildtype SARS-CoV-2, which is very different from that of current variants present in the population.

Researchers have successfully obtained high-affinity antibodies using antibody humanized mouse models in a few studies (33–36). Most of antibody humanized mice showed similar antibody repertoire with human. Hansen *et al.*, who used both humanized antibody mice Veloclmmune (VI mice) and COVID-19 patients to generate fully human antibodies (28). Next-generation sequencing was used to sequence the antibody variable regions. The results showed that most of VI mouse antibodies used VH3-53 paired with VK1-9, VK1-33 or VK1-39. Onodera *et al.* used humanized mice (TC-mAb mice) to identify an antibody that broadly neutralizes SARS-CoV-2 variants (37, 38). Analysis of transcripts revealed that the IGH and IGK transcripts in TC-mAb mice were similar with that in human peripheral blood monocytes. Nie *et al.* obtained several potent RBD-blocking antibodies from RenMab mice (33). They used the high-throughput single cell-based antibody sequencing approach and revealed that the IGHV3-30 subfamily was repeatedly used in RenMab mice and patients with COVID-19 (39). In contrast to humanized mice described above, SARS-CoV-2 neutralizing antibodies isolated from humanized mice (H2L2 mice) were derived from IGHV6-1 and IGKV4-1 germline gene (34, 40). Although a strong IGHV6-1 usage bias was found in CAMouse^HG^ mice, few IGHV6-1 paired with IGKV4-1 and most of IGHV6-1 paired with IGKV3-20 were revealed (**Figure 2**). Hence, these results suggest that combining CAMouse^HG^ approach with human platforms or other antibody humanized mice could allow the expanded capture of more diverse, potent neutralizing SARS-CoV-2 mAbs.

Hybridoma method and single B cell sorting method are the most commonly used to isolate active antibodies from humanized mice. Single B cell sorting method is quick and has high throughput but depends on the key protein of viral invasion (41). However, for a lot of mammalian viruses, the key proteins involved in viral entry are still unknown, especially in the early stage of the pandemic. This disadvantage is also obvious when single B cell sorting method was used to isolate antibodies from human (42–44). In contrast to single B cell sorting method, determining the key protein of viral invasion in hybridoma method is unnecessary. The supernatant of each hybridoma clone containing monoclonal antibody can be used directly for virus binding analysis and virus neutralization assay. However, generation of monoclonal hybridoma lines is time consuming. The existing routine immunization programs are usually scheduled once every 2-3 weeks. In general, up to 4 immunizations, which take at least 42-63 days, are needed to get high serum antibody titers. In this study, we performed a 28-day rapid immunization program, that was time saving. Moreover, generation of monoclonal hybridoma clones is time consuming. Chunyan Wang *et al.* select hybridomas using HAT medium and screened them by using antigen-specific ELISA. The positive hybridoma cells were subcloned to generate monoclonal hybridoma lines for further antigen-specific ELISA and other analysis (34, 40). In this study, to enable the rapid screening and discovery of SARS-CoV-2 reactive and neutralizing antibodies from humanized mice CAMouse^HG^, we utilized a type of semisolid medium containing HAT to generate monoclonal hybridoma lines by one step. The supernatant was collected for tests of antibody activity. Our approach greatly reduces experimental time.

In conclusion, we utilized humanized CAMouse^HG^ mice to develop a rapid antibody discovery method, analyzed the antibody repertoire of hybridoma cells of immunized CAMouse^HG^ mice and successfully generated a noncompeting two-antibody cocktail, which showed a potent prophylactic protective efficacy against SARS-CoV-2 in cynomolgus macaques. These results indicate that humanized CAMouse^HG^ mice not only provide a valuable platform to obtain fully human reactive and neutralizing antibodies, but also have a different antibody repertoire from human which can be used as a good complementary tool in the discovery of fully human therapeutic and diagnostic antibodies.

## Data availability statement

The datasets presented in this study can be found in online repositories. The names of the repository/repositories and accession number(s) can be found below: https://bigd.big.ac.cn/ under BioProject accession number CRA007302 and OMIX accession number OMIX001600.

## Ethics statement

The animal study was reviewed and approved by The Laboratory Animal Ethics Committee of ChongQing Academy of Animal Sciences and The Animal Welfare and Ethics Committee of the Changchun Veterinary Research Institute at the Chinese Academy of Agricultural Sciences.

## Author contributions

LG, XX, and TW conceived, designed and supervised the experiments. XY, HC, MW, and QL wrote the manuscript and carried out the data analysis. XY, HC, MW, ZW, QL, QH, XL, YL, XYW, NH, JB and HL performed the experiments. XWW performed the histopathology evaluation. YG, YZ, NF, and SY provided advice on study design. All of the authors have read and approved the final manuscript.

## Funding

This work was supported by National Natural Science Foundation of China (grant number 32000130); Jilin Province Youth Talent Support Project (grant number QT202115); Special Fund in Chongqing of Performance Incentive Guide (grant number cstc2021jxjl0017); Special key project of Chongqing technology innovation and application development (grant number cstc2020jscx-fyzxX0006); Special Projects in Agricultural and Animal Husbandry High-Tech Industry Research and Development of Chongqing Rongchang (grant number cstc2020ngzx0023).

## Conflict of Interest

Author MW is part-time employed by Chongqing Jinmaibo Bio. MW, LG and XL are shareholders of Chongqing Jinmaibo Bio.

The remaining authors declare that the research was conducted in the absence of any commercial or financial relationships that could be construed as a potential conflict of interest.

## Publisher’s note

All claims expressed in this article are solely those of the authors and do not necessarily represent those of their affiliated organizations, or those of the publisher, the editors and the reviewers. Any product that may be evaluated in this article, or claim that may be made by its manufacturer, is not guaranteed or endorsed by the publisher.

## Supplementary material

Supplementary Figure 1 Flow cytometry analysis of antibody binding activity to 293T cells transiently expressing SAR-CoV-2 S protein (293T/S) and untransfected 293T cells.
