# Supplementary figures and images for: Discovery and characterization of SARS-CoV-2 reactive and neutralizing antibodies from humanized CAMouseHG mice through rapid hybridoma screening and high-throughput single-cell V(D)J sequencing
Source: Front Immunol. 2022 Sep 23;13:992787. doi: 10.3389/fimmu.2022.992787 (PMC9545174; doi:10.3389/fimmu.2022.992787)

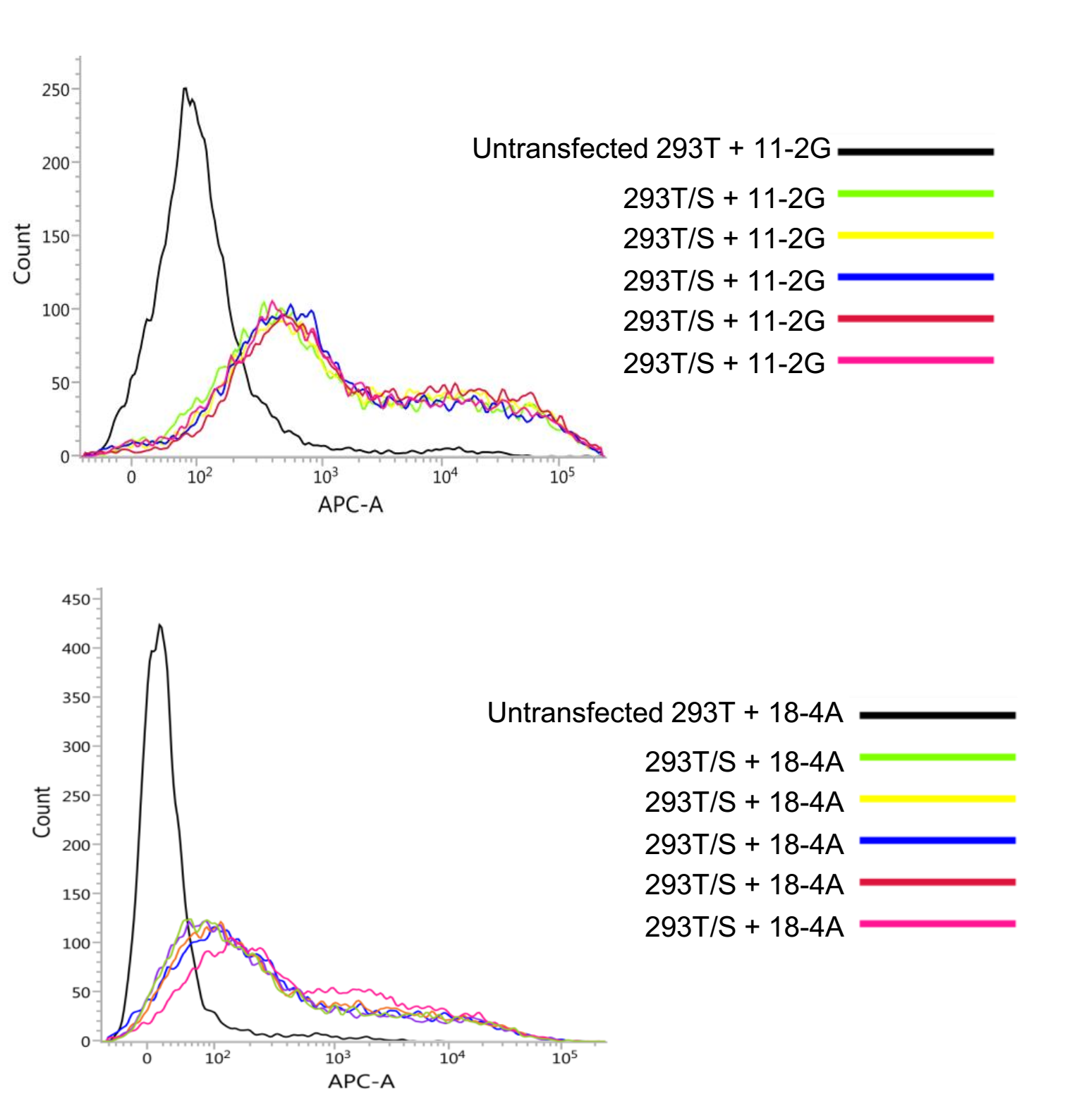

Supplement: Supplementary Figure 1 — Flow cytometry analysis of antibody binding activity to 293T cells transiently expressing SAR-CoV-2 S protein (293T/S) and untransfected 293T cells. [file Image_1.tif]
